# Effect of Cigarette Smoke Exposure and Aspirin Treatment on Neurotransmitters’ Tissue Content in Rats’ Hippocampus and Amygdala

**DOI:** 10.3390/metabo13040515

**Published:** 2023-04-04

**Authors:** Alaa M. Hammad, Ala A. Alhusban, Lujain F. Alzaghari, Fawaz Alasmari, Youssef Sari

**Affiliations:** 1 Department of Pharmacy, Faculty of Pharmacy, Al-Zaytoonah University of Jordan, Amman 11733, Jordan; ala.alhusban@zuj.edu.jo (A.A.A.);; 2 Department of Pharmacology and Toxicology, College of Pharmacy, King Saud University, Riyadh 11451, Saudi Arabia; ffalasmari@ksu.edu.sa; 3 Department of Pharmacology and Experimental Therapeutics, College of Pharmacy and Pharmaceutical Sciences, University of Toledo, Toledo, OH 43606, USA; youssef.sari@utoledo.edu

**Keywords:** dopamine, glutamate, serotonin, glutamine, GABA, anxiety-like behavior, aspirin

## Abstract

Cigarette smoke withdrawal can cause anxiety-like behavior and modulate neurotransmitter-related proteins in the brain. We examined the effects of cigarette smoke with and without aspirin treatment on the concentrations of neurotransmitters, including dopamine, serotonin, glutamate, glutamine, and GABA in the amygdala and hippocampus. Sprague-Dawley rats were randomly assigned to four different groups: (1) control group exposed only to standard room air, (2) cigarette smoke exposed group treated with saline vehicle, (3) cigarette smoke exposed group treated with aspirin (30 mg/kg), and (4) control group treated only with aspirin (30 mg/kg). Cigarette smoke exposure was performed for 2 h/day, 5 days/week, for 31 days. Behavioral testing was carried out weekly, 24 h after cigarette smoke exposure, during acute withdrawal. At the end of week 4, rats were given either distilled water (1 mL) or aspirin 45 min before cigarette exposure for 11 days. Dopamine, serotonin, glutamate, glutamine, and GABA were extracted from both the amygdala and hippocampus and were separated and quantified using a developed and validated HPLC-MS/MS method. Cigarette smoke withdrawal induced anxiety behaviors, and aspirin treatment reduced this effect. Cigarette smoke exposure increased tissue content of dopamine, serotonin, glutamate, glutamine, and GABA, and aspirin treatment reversed this effect. Cigarette smoke caused an increase in tissue content of several neurotransmitters as well as anxiety-like behavior, and these effects were normalized by aspirin treatment.

## 1. Introduction

Tobacco smoking has high prevalence, with 1.3 billion smokers worldwide as of 2022. Each year, more than eight million individuals die from tobacco use disorders. Around 1.2 million of those fatalities are non-smokers being exposed to secondhand smoke, while more than 7 million are caused by direct tobacco use [1]. Exposure to cigarette smoke is linked to anxiety disorders. Tobacco smokers experience greater anxiety than non-smokers [2], and quitting smoking is linked to higher levels of stress and anxiety because nicotine has been found to decrease anxiety state [3]. Indeed, a previous study reported that one of the coping approaches for stress among nurse students was smoking cigarettes [4]. There is evidence that people who are more anxious are more inclined to smoke tobacco [5]. The use of cigarettes has been suggested to be associated with self-medication to manage anxiety and to greater propensity to start smoking as a result of societal pressure [6]. However, studies suggested that smoking may increase the likelihood of experiencing higher anxiety [7,8,9]. Tobacco smoking’s effects on neurotransmitters, respiratory, and autonomic systems [10], in addition to its effects on normal neurodevelopmental processes, are potential explanatory models for anxiety [11]. Smoking and anxiety have a complicated association because there is evidence that some smokers can experience a reduction in anxiety (for more details see review [12]). Additionally, after quitting smoking, smokers frequently report feeling more anxious [13]. Several neurotransmitter systems play important roles in the pathogenesis of mood and anxiety disorders [14,15]. Smoking has a significant effect on the concentrations of neurotransmitters in the brain [16]. Nicotine affects the release of neurotransmitters such as dopamine, norepinephrine, and acetylcholine [10,16]. Prior studies demonstrated that elevation of extracellular neurotransmitters, including dopamine and glutamate, was linked to neurobehavioral changes in animals exposed to substances of abuse, including nicotine [17,18]. Dopamine, in particular, is strongly linked to the rewarding and reinforcing effects of smoking, and nicotine increases dopamine release in the synaptic cleft [19]. This leads to increased well-being feelings, reward, and reinforcement, which contribute to the development of nicotine dependence [20]. Smoking also affects the concentrations of other neurotransmitters such as serotonin, which is involved in regulating mood, and GABA, which is involved in regulation of anxiety, and glutamate as an excitatory neurotransmitter [3,11,21]. These changes in neurotransmitter concentrations can contribute to behavioral and psychological effects of smoking and play a role in the development of smoking-related disorders such as nicotine addiction [22].

The elevated plus maze (EPM) is a widely used tool for preclinical research in the field of anxiety disorders, as it provides a quick and easy way to assess anxiety-like behavior in rats. The EPM works on the principle that rodents have an innate fear of open spaces and prefer to stay in enclosed areas. Rats that are more anxious are expected to spend less time in the open arms and more time in the enclosed arms of the maze. The time spent in each arm is recorded, as well as the number of entries into each arm (for more details see review [23]).

The amygdala and hippocampus are two important brain regions that play a role in regulating emotion, particularly anxiety [24]. Note that activation of the amygdala is associated with increased anxiety-like behavior [25]. Damage to the hippocampus resulted in increased anxiety-like behavior, as well as impairments in spatial memory and stress regulation [25]. The hippocampus is known to have direct connections to the amygdala, and both regions regulate fear and anxiety responses [26]. Thus, in this study, we studied the effect of two hours per day exposure for five days per week for 31 days on anxiety and tissue content of several neurotransmitters in hippocampus and amygdala.

**Figure 1:**
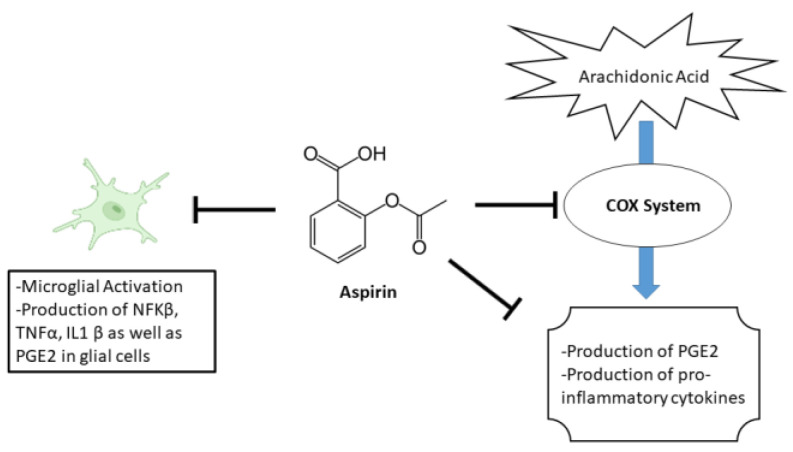
Illustration of aspirin’s mechanism.

Aspirin, a commonly used as nonsteroidal anti-inflammatory drug (NSAID), has been suggested to have potential effects on anxiety [27]. The primary mechanism of aspirin’s effect is through its inhibition of the enzyme cyclooxygenase (COX). Specifically, aspirin blocks the enzyme’s ability to convert arachidonic acid into prostaglandins. This leads to a decrease in the production of various pro-inflammatory cytokines as well as PGE2 [28]. In addition to its effects on COX, aspirin also has other biochemical effects that may contribute to its therapeutic effects. For example, aspirin has been shown to inhibit the activation of the transcription factor NF-κB, which is involved in the regulation of genes that are involved in inflammation and immune response. Furthermore, aspirin reduces hippocampal inflammatory marker cytokines IL-1β, TNF-α, and brain PGE2 levels [29,30]. An illustration of aspirin’s mechanism is shown in Figure 1.

Although aspirin is primarily used for pain relief and inflammation reduction, studies have found that it can also reduce anxiety state in certain individuals [31]. However, aspirin is not a selected drug to treat anxiety disorders, and its effects on anxiety may be related to its anti-inflammatory and analgesic benefit as mode of action. Indeed, aspirin has shown to have an effect on mental health through its potential ability to reduce the risk of developing Alzheimer’s disease [32] and reduce symptoms of depression [33], schizophrenia [34], and other mental illnesses [35]. Importantly, aspirin has been shown to have an effect on certain neurotransmitters, although the exact mechanism is not well understood [36]. It is believed that aspirin may affect neurotransmitter concentrations by reducing inflammation and oxidative stress in the brain, both of which have been linked to changes in neurotransmitter concentrations in the synaptic cleft [37]. Studies have found that aspirin can increase the concentrations of serotonin and norepinephrine, and these neurotransmitters are involved in regulating mood, while also reducing levels of prostaglandins, which have been linked to pain and inflammation [38]. Additionally, aspirin has an effect on the concentration of acetylcholine, which is involved in learning and memory [39]. Indeed, several studies have shown that aspirin can upregulate astroglial glutamate transporter (GLT-1) expression in the brain [40,41]. A previous study has reported that treatment with aspirin increased GLT-1 expression in the hippocampus [42], nucleus accumbens, and prefrontal cortex [41]. The exact mechanism by which aspirin upregulates GLT-1 is not fully understood, but it is thought to involve the activation of certain signaling pathways, including the nuclear factor kappa B (NF-κB) pathway [43]. However, aspirin may also reduce inflammation, which could indirectly affect GLT-1 expression and activity [44]. The effects of aspirin on neurotransmitter concentrations are complex and not fully understood, and may vary depending on factors such as dose, duration of use, and individual factors. The effects of aspirin on neurotransmitter concentrations may not necessarily translate to an improvement in mood symptoms, and further research is needed to fully understand the mechanisms by which aspirin affects neurotransmitter concentrations in the brain [36]. Therefore, in this study, we investigated the effect of aspirin on anxiety-like behavior and investigated the associated changes in the level of several neurotransmitters such as dopamine, serotonin, glutamate, glutamine, and GABA in the hippocampus and amygdala.

## 2. Materials and Methods

### 2.1. Animals

Twenty female Sprague-Dawley rats weighing 180–250 g at the age of 10–12 weeks were inbred in animal room facility with 50% ± 2% room humidity and temperature of 21 °C ± 2 °C at Al-Zaytoonah University of Jordan (ZUJ). Sawdust was utilized as bedding for rats. All experimentations were performed during the light cycle in which the light–dark cycle was 12 h. The experimental and housing techniques were conducted in accordance with the Helsinki guidelines for animal research [45], and all procedures were approved by the Institutional Animal Care and Use Committee at ZUJ. Animals were randomly distributed into four groups following baseline EPM measurements: (a) the control group exposed to room air throughout the study with a distilled water oral gavage for the last eleven days of the study (*n* = 5); (b) the NIC group exposed to a whole body cigarette throughout the experiment with a distilled water oral gavage treatment for the last eleven days of the study (*n* = 5); (c) the NIC/aspirin group exposed to a whole body cigarette group with oral gavage of aspirin (30 mg/kg) for the last eleven days of the study (*n* = 5); and (d) the aspirin group exposed to room air with oral gavage of aspirin (30 mg/kg) for the last eleven days of the study (*n* = 5). All groups had free access to food and regular tap water during the experiments.

### 2.2. Chemicals and Materials

Dopamine hydrochloride, γ-aminobutyric acid (GABA), serotonin hydrochloride, glutamine, glutamate, epinephrine (internal standard, IS), formic acid, and heptafluorobutyric acid (HFBA) were obtained from Sigma-Aldrich (St. Louis, MO, USA). Trifluoroacetic acid (TFA) was acquired from Fisher Scientific (Loughborough, Leics, UK). LC/MS grade water, methanol, and acetonitrile were purchased from Honeywell Burdick and Jackson (Muskegon, MI, USA). Milli-Q water was supplied by a Milli-Q^®^ water purification system (Millipore, Bedford, MA, USA). Liggett Ducat blue cigarettes cruise (LD, 0.6 mg of nicotine, 0.8 mg of tar, and 0.01 mg of carbon monoxide) were purchased from the local market.

### 2.3. Cigarette Smoke Exposure

**Figure 2:**
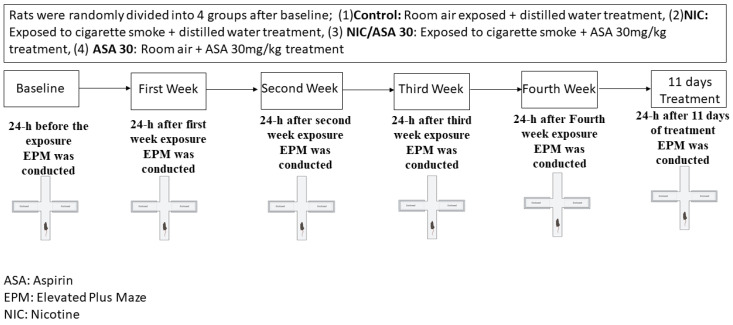
Experimental timeline for cigarette exposure, aspirin treatment, and EPM testing. Rats were randomly divided into four groups after baseline; (1) control: room air exposed + distilled water (1 mL) treatment, (2) NIC: exposed to cigarette smoke + distilled water (1 mL) treatment, (3) NIC/aspirin: exposed to cigarette smoke + aspirin 30 mg/kg treatment, (4) aspirin: room air + aspirin 30 mg/kg treatment.

The timeline of exposure to cigarette smoke, aspirin treatment, and behavioral assessments is represented in Figure 2. Despite the fact that the exposure to cigarette smoke was passive through a whole-body exposure, the rates of exposure more closely resemble those produced by active smoking, and the effects are suggested to be predominantly mediated by inhalation route. Nicotine groups were exposed to tobacco cigarettes for 2 h/day, 5 days/week for four weeks. Rats received an oral gavage of either distilled water (1 mL) or aspirin (30 mg/kg) every day 45 min prior to exposure to tobacco cigarettes for 11 consecutive days. Rats were placed inside an acrylic exposure chamber (40 × 40 × 40 cm), which had a door in the upper portion, and the chamber was subsequently sealed throughout the exposure to tobacco smoke. Three air vents were at the top of the door for air circulation, and there was a fourth opening for the tobacco cigarette exposure. A pump was attached to the exposure chamber, and was altered to permit the circulation of tobacco smoke through a tube. The pump controller was attached to a timer that regulates the puff duration and inter-puff intervals. For the length of the exposure, repeated cycles of a three second puff with a thirty second inter-puff interval were used in this experiment. Two cigarettes were burnt simultaneously to saturate the exposure chamber before the rats were placed inside, and then the standard cycles of tobacco cigarette smoke exposure were initiated. In the course of the two hours of exposure, twelve cigarettes were smoked. An electrochemical sensor (Monoxor II, Bacharach Inc., New Kensington, PA, USA) was used to measure the amounts of carbon monoxide (CO) in the exposure chamber. The CO concentration was maintained at 700 ppm or below during the exposure procedure. Additional cigarettes were therefore used if CO concentrations were below 500 ppm, and the pump was turned off for 1–2 min to return the desired CO concentration if it was higher than 899 ppm.

### 2.4. Elevated plus Maze Test

The most common and basic behavioral test used to assess anxiety-like behavior in rats is the EPM [46]. The EPM was made up of four cross-shaped arms with a central zone in the middle that is about 50 cm above the ground. The EPM consisted of two open arms (without walls; 50 cm × 10 cm; L × W) and two closed arms (with black color walls; 50 cm × 10 cm × 30 cm; L × W × H). Each rat was placed at the crossroad of the elevated plus maze’s four arms, facing the open arm, to begin the 5 min test. A camera 110 cm above the ground was used to record the experiment for later behavioral study. Following each rat test, the maze was cleaned with distilled water. A weak light illuminated the testing area (25 W). Importantly, the following behaviors were measured: time spent in an open arm as well as number of crossings.

### 2.5. Brian Regions Collection

Following the last EPM measurement, rats were quickly euthanized using diethyl ether (Sigma-Aldrich, St. Louis, MO, USA) inhalation, and then decapitated using a guillotine. Brains were then removed, flash frozen in liquid nitrogen, and kept at −80 °C for further analysis. Following, whole brains were then taken from −80 °C freezer and using a cryostat (Leica Biosystems, Deer Park, IL, USA), the hippocampus and amygdala were located, sectioned using the Rat Brain Atlas [47], and the tissues were kept at −80 °C for subsequent experiment.

### 2.6. Sample Preparation and Chromatographic Analysis

**Figure 3:**
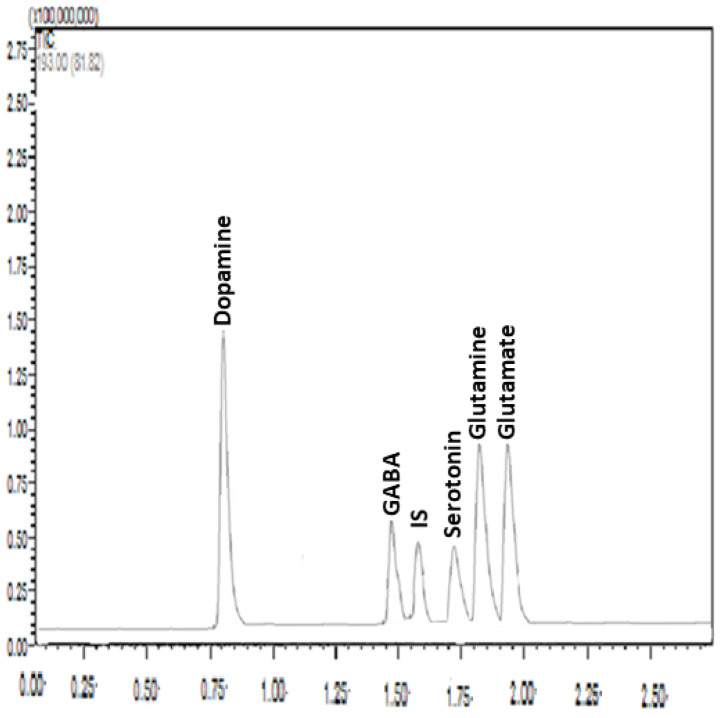
A representative chromatogram under optimized conditions: Zorbax SB C18 column (3.0 × 100 mm, 1.8 μm particle size); mobile phase, 0.3% heptafluorobutyric acid (HFBA) and 0.5% formic acid solution–acetonitrile with gradient elution; flow rate, 0.3 mL/min; and injection volume, 5 μL. The analytes of interest are shown above each peak. IS: Internal standard (epinephrine), X axes (retention time in minutes), Y axes (relative intensity).

Samples from the hippocampus and amygdala were homogenized in 100 µL of Milli-Q water. Homogenization was carried out in an ice bath to prevent the degradation of the analytes. The samples were first homogenized using a vortex mixer, and then they were mixed with 20 µL TFA in a fume hood to precipitate protein. The mixture was vortexed and centrifuged at 15,000 rpm for 20 min at 4 °C. The internal standard (IS) solution was added to 50 μL of the supernatant, which was then well mixed before being filtered through a 0.22 μm syringe filter. The HPLC-MS/MS equipment was loaded with a 5 μL aliquot. Tissue content of neurotransmitters including glutamate, glutamine, GABA, dopamine, and serotonin was measured accurately and precisely following the developed and validated method as described in detail in our previous study [48] (Figure 3). Briefly, liquid chromatography separation was implemented using a Shimadzu Nexera X2 UHPLC system equipped with a Zorbax SB C18 column (3.0 × 100 mm, 1.8 μm particle size; Agilent) at a controlled column temperature of 25 °C. The mobile phase consisted of HPLC-grade water and acetonitrile, each containing 0.3% HFBA and 0.5% formic acid at gradient conditions. Elution was carried out at a flow rate of 0.3 mL/min. HPLC–MS/MS analysis was conducted using a Shimadzu LC-8030 triple quadrupole equipped with an electrospray ionization (ESI) source detector. MS parameters were as follows: 300 C gas temperature, 7 L/min drying gas, 50 psi nebulizer pressure, 325 C sheath gas temperature, 10 L/min sheath gas flow, 3750 V capillary voltage, 20 ms dwell time, and 0 V nozzle voltage. A sample for the chromatograms under the optimized conditions is presented in Figure 3.

### 2.7. Sample Size Calculation

Sample size was calculated using two methods; a resource equation method, in which E = Total number of animals − Total number of groups. E is the degree of freedom of analysis of variance (ANOVA). The value of E should lie between 10 and 20 [49]. In our study E = 16. The second method was carried out using the Gpower statistical program, in which we used an effect size of f = 1.2, α-errors = 0.05, and a power of 0.95 for four groups and the total number of animals was 20.

### 2.8. Statistical Analysis

Data were presented as means and standard errors of the means (SEM). Two-way repeated measures ANOVA, followed by Tukey’s multiple comparisons, were used to analyze EPM results. One-way ANOVA followed by Tukey’s multiple comparisons was used to evaluate neurotransmitters’ tissue content. All statistical analyses were based on a *p* < 0.05 level of significance using GraphPad Prism version 9.0.

## 3. Results

### 3.1. Effect of Whole-Body Tobacco Cigarette Smoke Exposure and Aspirin Treatment on Elevated plus Maze Behavioral Test

**Figure 4:**
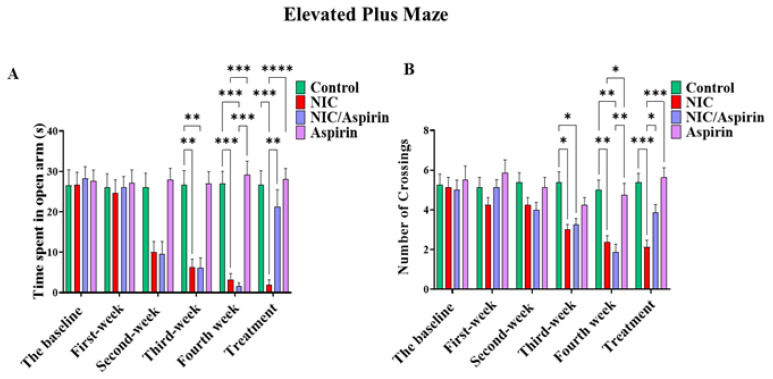
Behavioral testing in the elevated plus maze (EPM) test. (**A**) Time spent in open arm in the EPM in control, NIC, NIC/aspirin, and aspirin groups; (**B**) number of crossings in the EPM in control, NIC, NIC/aspirin, and aspirin groups. Data are presented as mean ± SEM (* *p* < 0.05, ** *p* < 0.01, *** *p* < 0.001, **** *p* < 0.0001; *n* = 5 for each group).

Withdrawal-induced anxiety resulting from whole-body tobacco cigarette smoke exposure was observed after the second week of exposure as indicated by the time spent in the open arms (Figure 4A), and in third week as shown with the number of crossings (Figure 4B). This effect was reversed by 11 doses of 30 mg/kg of aspirin, and it was confirmed by repeated measures two-way ANOVA, which revealed a significant main effect of Time [F (3, 16) = 41.09, *p* < 0.0001], a significant effect of Treatment [F (3, 16) = 9.120, *p* = 0.0002], and a significant Time × Treatment interaction [F (15, 80) = 19.78, *p* < 0.0001]. Tukey’s multiple comparisons test showed a significant decrease in time spent in open arm starting from week 2 in the NIC and NIC/aspirin groups relative to the control and aspirin groups. After 11 days of treatment, the NIC group exhibited a significant difference in time spent in open arms compared with the NIC/aspirin, aspirin, and control groups (Figure 4A).

A parallel pattern of effects was observed in the number of crossings. Repeated measures two-way ANOVA revealed a significant main effect of Time [F (3, 16) = 14.28, *p* < 0.0001], a significant effect of Treatment [F (3, 16) = 6.939, *p* = 0.0012], and a significant Time × Treatment interaction [F (15, 80) = 3.949, *p* < 0.0001]. Tukey’s multiple comparisons test confirmed a significant difference between the NIC and NIC/aspirin groups compared with the control and aspirin groups at weeks 3 and 4. This effect was attenuated after treatment for 11 days with aspirin 30 mg/kg; Tukey’s multiple comparisons revealed a significant difference in the number of crossings in the NIC group compared with NIC/aspirin, aspirin, and control groups (Figure 4B).

### 3.2. Effect of Whole-Body Cigarette Smoke Exposure and Aspirin Treatment on Tissue Content of Neurotransmitters in Hippocampus

**Figure 5:**
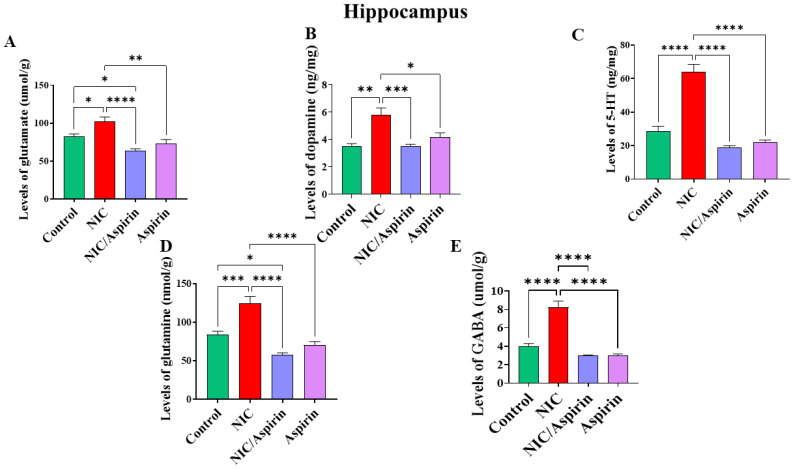
Tissue content of neurotransmitters after tobacco cigarette smoke exposure and aspirin treatment in the hippocampus. (**A**) Glutamate; (**B**) dopamine; (**C**) serotonin; (**D**) glutamine; and (**E**) GABA. Data expressed as mean ± SEM (* *p* < 0.05, ** *p* < 0.01, *** *p* < 0.001, **** *p* < 0.0001; *n* = 5 for each group).

Whole-body cigarette smoke exposure for 31 days showed a significant elevation in neurotransmitters’ tissue content in the hippocampus, and treatment with aspirin 30 mg/kg for 11 days normalized this effect (Figure 5). One-way ANOVA revealed a significant main effect of treatment on the tissue content of glutamate in the hippocampus [F (3, 16) = 13.24, *p* = 0.0001; Figure 5A]. A significant main effect of treatment was observed on tissue content of dopamine in the hippocampus [F (3, 16) = 13.40, *p* = 0.0001; Figure 5B]. In addition, a significant main effect of treatment was observed on the tissue content of serotonin in the hippocampus [F (3, 16) = 54.49, *p* = 0.0001; Figure 5C]. A significant main effect of treatment was also observed on the tissue content of glutamine in the hippocampus [F (3, 16) = 25.54, *p* < 0.0001; Figure 5D] and on the tissue content of GABA in the hippocampus [F (3, 16) = 42.51, *p* < 0.0001; Figure 5E].

### 3.3. Effect of Whole-Body Cigarette Smoke Exposure and Aspirin Treatment on Tissue Content of Neurotransmitters in the Amygdala Brain Region

**Figure 6:**
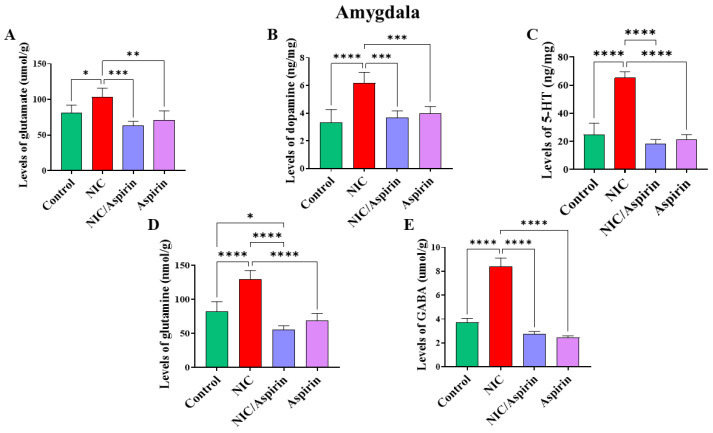
Tissue content of neurotransmitters after tobacco cigarette smoke exposure and aspirin treatment in the amygdala. (**A**) Glutamate; (**B**) dopamine; (**C**) serotonin; (**D**) glutamine; and (**E**) GABA. Data expressed as mean ± SEM (* *p* < 0.05, ** *p* < 0.01, *** *p* < 0.001, **** *p* < 0.0001; *n* = 5 for each group).

Whole-body cigarette smoke exposure for 31 days showed a significant increase in neurotransmitters’ tissue content in the amygdala, and treatment with aspirin 30 mg/kg for 11 days normalized this effect (Figure 6). One-way ANOVA revealed a significant main effect of treatment on tissue content of glutamate in the amygdala [F (3, 16) = 13.17, *p* = 0.0001; Figure 6A]. A significant main effect of treatment was observed on the tissue content of dopamine in the amygdala [F (3, 16) = 16.40, *p* < 0.0001; Figure 6B]. A significant main effect of treatment in was also observed on the tissue content of serotonin in the amygdala [F (3, 16) = 89.67, *p* < 0.0001; Figure 6C]. Furthermore, a significant main effect of treatment was observed on the tissue content of glutamine in the amygdala [F (3, 16) = 39.33, *p* < 0.0001; Figure 6D] and on the tissue content of GABA in the amygdala [F (3, 16) = 41.36, *p* < 0.0001; Figure 6E].

## 4. Discussion

We previously reported that tobacco cigarette smoke exposure induced changes in the content of neurotransmitters in the hippocampus and amygdala [48]. In addition to these findings, we reported here that rats exposed to tobacco cigarette smoke developed anxiety-like behavior. We also demonstrated that aspirin at a dose of 30 mg/kg restored the tissue content of these neurotransmitters. Moreover, aspirin showed the ability to decrease anxiety-like behaviors.

Previous studies have revealed that diminishing in repetitive behavior and sociability (autistic behavior) were found in animals exposed to oral consumption of nicotine [50], and increased behavioral sensitization in mice exposed to low dose of nicotine-containing electronic cigarette [51]. Moreover, exposure to electronic cigarette containing nicotine (25 mg/mL) was found to induce alterations in memory recognitions measured by increased number of entries into the novel object zone [52]. In addition, studies reported increase in locomotion activities in animals exposed to electronic cigarette containing nicotine [52] and a decrease in locomotor activities in waterpipe tobacco smoke exposure [53]. In agreement with a previous study from our laboratory, we found that exposure to nicotine-containing waterpipe induced anxiety-like behaviors starting in the second week through the end of the study using elevated plus maze test [53]. These behavioral changes include time spent in the open arms and number of crossings. Aspirin (30 mg/kg) normalized these changes in anxiety-like behaviors. In accordance, studies from our lab showed that aspirin reduced cigarette smoke-induced anxiety-like behavior and this effect was associated in part with the normalization of GLT-1 expression [41]. In fact, the normalization of GLT-1 might be associated with the normalization of extracellular glutamate concentration in key reward regions in the brain [54].

Extracellular concentration of glutamate is a key player in the development of nicotine craving, withdrawal, and reinstatement [55,56]. Several studies reported that intermittent nicotine exposure either orally [57] or through electronic delivery was associated with reduced expression of astroglial glutamate transporter, including GLT-1 in certain brain regions [58,59]. In addition, cystine/glutamate exchange transporter (xCT) expression was found to be reduced in the hippocampus of mice exposed to electronic cigarettes containing nicotine, and rats orally self-administered nicotine for 4 weeks or electronic cigarette vapors containing nicotine for several months [57,58,59], which regulate glutamate release via metabotropic glutamate receptor (mGluR2/3) activation. Therefore, the reduction of GLT-1 expression is associated with an increase in extracellular glutamate concentrations in the mesocorticolimbic system. Previous studies from our lab showed that aspirin (30 mg/kg) normalized the expression of GLT-1 and xCT in rats exposed to cigarette smoke containing nicotine for four weeks [41]. Moreover, a previous study reported an increase in extracellular glutamate concentrations in the frontal cortex following 6-month exposure to electronic cigarettes containing 24 mg/mL nicotine in mice [60]. It is important to note that nicotine may stimulate the release of glutamate in part by activating nicotinic acetylcholine receptors (nAChRs), primarily the alpha-7 subtype of nAChR (α-7 nAChR) in the frontal cortex, an effect abolished with a selective α-7 nAChR blocker [61,62]. Although the hippocampus and amygdala send glutamatergic inputs to other brain areas, they receive glutamatergic projections from the frontal cortex [63]. Importantly, a previous study also indicated that glutamate concentrations are increased after chronic exposure to nicotine [60], similar to the results of our present study. Moreover, our work found that aspirin, an astroglial glutamate transporter upregulator, could modulate these effects.

The glutamate–glutamine cycle involves enzymes that convert glutamate to glutamine [64]. In addition to glutamate, this present study revealed that tissue content of glutamine was increased in hippocampus and amygdala of mice exposed to tobacco cigarette smoke. It important to note that 6-month exposure to nicotine-containing electronic cigarette was associated with increased in tissue content of glutamine in the frontal cortex and striatum [60]. Moreover, glutamine biosynthesis was found to be increased following 4-week treatment with nicotine through a subcutaneous route of administration [65]. Our present results revealed that glutamine was elevated in the hippocampus and amygdala in rats exposed to nicotine, and aspirin attenuated this effect. Our findings indicate that aspirin might be a potential compound for normalizing the glutamate–glutamine cycle after exposure to tobacco cigarette products.

Nicotine increases dopamine neurotransmission at least in part through modulating nAChRs in the brain [66]. Pre-clinical studies demonstrated that nicotine exposure was associated with elevated extracellular dopamine concentrations in the nucleus accumbens and ventral tegmental area [67]. However, chronic inhalation of electronic cigarette vapors containing 24 mg/mL nicotine was associated with decreased in tissue content of dopamine in striatum [60]. In addition, chronic nicotine exposure reduced the release of dopamine in the nucleus accumbens [68]. Our present study suggests that shorter duration of exposure to nicotine (4 weeks) is associated with increased in tissue content of dopamine in the hippocampus and amygdala. Our findings are in accordance with a previous study reporting that dopamine concentration in the caudate putamen was increased in rats treated with nicotine (0.125 mg/kg, s.c.) for 2 weeks [69]. Interestingly, aspirin normalized the tissue content of dopamine in the hippocampus and amygdala after nicotine treatments for 4 weeks. Together, aspirin was able to restore the tissue content of glutamate and dopamine in animal models of tobacco cigarette smoke exposure.

Our present findings revealed that 4-week cigarette smoke containing nicotine delivery increased tissue content of GABA in the hippocampus and amygdala. Accordingly, 6-month inhalation of electronic cigarette vapors-containing 24 mg/mL nicotine was associated with reduced tissue content of GABA in the frontal cortex of mice [60]. It is important to note that study showed that GABA biosynthesis was increased in the brain cortex region in mice treated with nicotine (2 mg/kg, s.c.) for 4 weeks [65]. These data suggest an increase in the overall GABA concentrations in the mesocorticolimbic brain regions with nicotine exposure. Further studies are warranted to investigate the effects of chronic exposure to nicotine on GABA biosynthesis in different brain areas with respect to the duration of tobacco cigarette smoke exposure. It is noteworthy to mention that GABA can be produced by glutamate metabolism. The increases in tissue content of GABA in the amygdala and hippocampus were restored with aspirin, indicating that this latter is considered a potential compound that may increase GLT-1 expression and consequently reduced extracellular glutamate concentration in the synaptic cleft, and normalize imbalance in neurotransmitters (glutamate and GABA) that was caused by nicotine exposure.

Our study showed that 4-week exposure to nicotine through cigarette tobacco smoke increased tissue content of serotonin in the hippocampus and amygdala, and that aspirin modulated these alterations. In accordance, studies reported that serotonin system is affected by chronic exposure to nicotine [70,71]. It was shown that the release of serotonin in the striatum, but not in the prefrontal cortex, was increased in stressed rats treated with nicotine (0.4 mg/kg, i.p) for 2 weeks [71]. In addition, another study showed that the reuptake of serotonin in the hippocampus and prefrontal cortex was increased in animals treated with nicotine (0.7 mg/kg, s.c. twice a day) for 10 days [72]. In addition, another study found that gene expression of serotonin transporters in the dorsal raphe was decreased with nicotine treatments (6 mg/kg/day, minipumps) for 12 days [70].

In conclusion, aspirin not only acts on a single neurotransmitter, but it can modulate multiple neurotransmitters in our established model of tobacco cigarette smoke. These effects provide evidence about the possible role of aspirin in the attenuation of tobacco-seeking behaviors. Our results suggest that there are potential therapeutic benefits of aspirin for normalizing the concentrations of several neurotransmitters in the brain and possibly might be considered for the treatment of tobacco use disorders, withdrawal, relapse, and craving. Studies are warranted to investigate the potential therapeutic effects of aspirin in animal models, including electronic and conventional cigarettes, pipes, hookah, and other nicotine and tobacco delivery systems.

## Data Availability

Data sharing not applicable.
